# Efficacy of vitamin D supplementation on depressive symptoms in older patients: a meta-analysis of randomized controlled trials

**DOI:** 10.3389/fmed.2024.1467234

**Published:** 2024-10-10

**Authors:** Jiamin Fu, Yuchi Zhang, Xiaoyu Chen, Xing Yu, Maoxin Yan, Biying Jing, Hongjuan Yu, Wenzhen Li, Qi Guo

**Affiliations:** ^1^Department of Rehabilitation Medicine, Shanghai University of Medicine and Health Sciences Affiliated Zhoupu Hospital, Shanghai, China; ^2^School of Sports and Health, Tianjin University of Sport, Tianjin, China; ^3^Department of Rehabilitation Medicine, Shanghai University of Medicine and Health Sciences, Shanghai, China; ^4^Cardiac Rehabilitation Center, Department of Cardiovascular Medicine, Inner Mongolia People’s Hospital, Hohhot, Inner Mongolia, China

**Keywords:** geriatrics, depression, depressive symptoms, vitamin D, therapeutic evaluation, meta-analysis

## Abstract

**Background:**

The relationship between vitamin D and depression has garnered significant attention in recent years. However, the efficacy of vitamin D in ameliorating depression among specific subgroups of older patients remains controversial. This study aimed to assess the impact of vitamin D supplementation on depressive symptoms and the prevalence of depression in older adults. Additionally, the study sought to examine potential moderating factors, including differences among population subgroups and various supplementation strategies.

**Methods:**

A systematic literature search was conducted in the databases PubMed, EMBASE, Web of Science, and the Cochrane Library up to March 2024. The RevMan 5.3 software was utilized to calculate the standardized mean difference (SMD) and to evaluate the quality of evidence using the Grading of Recommendations Assessment, Development and Evaluation (GRADE) approach. The objective was to determine the efficacy of vitamin D supplementation in alleviating depressive symptoms or treating depression in older adults.

**Results:**

This meta-analysis encompassed eleven studies, comprising a total of 21,561 participants. The findings did not indicate a statistically significant therapeutic benefit of vitamin D supplementation for depression in older patients [SMD: −0.10; 95% CI: (−1.19, 0.00); *p* = 0.05]. Subgroup analyses revealed that the efficacy of vitamin D intervention in geriatric depression correlated with several factors, including baseline serum 25(OH)D levels, the dosage of the intervention, gender, and the initial presence of depressive symptoms or a diagnosis of depression.

**Conclusion:**

The current evidence is insufficient to conclusively establish the significant efficacy of vitamin D supplementation in alleviating depressive symptoms among older patients. Consequently, additional randomized controlled trials are warranted to further validate the relationship between vitamin D supplementation and depression in the older adults.

## 1 Introduction

Depression is a serious mental illness with an estimated global prevalence of 4.4% (1). Depression is anticipated to become the predominant contributor to the global burden of disease and morbidity by the year 2030 (2). A variety of biological and psychosocial factors contribute to the condition. Biological factors include cerebrovascular damage, reduced volume in key brain regions, cognitive decline, and an increase in somatic and chronic comorbidities. Psychosocial factors encompass environmental mastery, life goals, autonomic life stressors, social relationships, and general life stressors (3), growing older has been identified as a risk factor for depression (4, 5). The swift expansion of the older population, defined as individuals over the age of 65, is contributing to an escalating prevalence of mental health disorders among this demographic. This trend is emerging as a significant public health concern. Specifically, the estimated prevalence of depression within this age group ranges from 5 to 15% (6). The prevalence of depression and suicide among individuals aged 65 and older is significantly higher compared to other age groups, resulting in a substantial decrease in quality of life and elevated mortality rates (3). However, geriatric depression is often overlooked in therapeutic practice, leading to occasional instances of underdiagnosis or undertreatment.

Traditional psychotherapy and pharmacological interventions remain the cornerstone of depression treatment, often supplemented by electroconvulsive therapy and exercise therapy. These treatments are frequently combined to enhance their efficacy. However, these conventional approaches may not be optimal for older patients with depression due to the presence of multiple comorbidities and factors such as reduced cognitive function and physical activity levels (7), and adherence to antidepressant treatment is generally poor due to several adverse reactions to antidepressant medications and the complexity and difficulty of performing treatments such as exercise therapy (8). The importance of vitamin D for overall mental health has garnered attention, prompting suggestions that dietary interventions aimed at achieving adequate vitamin D intake may offer a more accessible, convenient, and compliant alternative to traditional treatments. These interventions are particularly relevant given the challenges associated with managing depression in older adults, who often face multiple comorbidities and age-related declines in cognitive function and physical activity.

In addition to being created in the skin by exposure to sunshine, vitamin D can also be obtained through diet and supplementation. Regarded as a crucial nutrient for the body’s ability to absorb calcium and maintain calcium homeostasis, it plays a crucial role in bone metabolism (9). Vitamin D levels in the body are generally reflected by serum 25-hydroxyvitamin D (25(OH)D) levels, and there is no consensus on its prescribed thresholds and supplementation recommendations. Variations among guidelines often arise due to the distinct populations they aim to serve and the diverse approaches employed in evidence synthesis (10). This study used a clinical practice guideline published by the American Endocrine Society in 2011 as this guideline focuses more on those at high risk for vitamin D deficiency and would be more relevant to vitamin D research in the older population. In accordance with the 2011 American Endocrinology clinical practice guideline, we utilized the specified 25(OH)D levels as benchmarks: levels of 30 ng/mL or higher are deemed beneficial for bone health, 20–30 ng/mL indicate vitamin D insufficiency, and below 20 ng/mL signify vitamin D deficiency. This guideline is particularly relevant for older adults, who are at an increased risk of experiencing vitamin D deficiency (11). Alarmingly, vitamin D deficiency is expected to affect approximately one billion people worldwide (12, 13), and it is estimated that approximately 30% of children and 60% of adults worldwide are vitamin D insufficient or deficient (14). The effects of vitamin D on the extraskeletal system have gained a lot of attention in recent decades. Researchers have discovered that, although there may be some disagreement (15), vitamin D deficiency or insufficiency may contribute to the development of a variety of diseases affecting the immune, neurological, and cardiovascular systems as previously mentioned (16).

In recent years, the influence of vitamin D on brain function has become more evident, leading to a growing body of research that links vitamin D to the pathophysiology of depression. Studies have indicated that vitamin D supplementation may exert a protective effect against depression in mentally healthy adults, according to a meta-analysis conducted by Guzek (17) and Xie (18), who also discussed the therapeutic or preventive benefits of vitamin D on depression in these individuals. According to Spedding, vitamin D supplementation has been observed to reduce depressive symptoms and lower depression scale scores in both depressed and non-depressed mixed populations (19) and Shaffer (20) investigations. However, paradoxes have been raised in recent years by Gowda, Guzek (21), and Li (22), who conducted meta-analyses of vitamin D treatment in depressed adults, and tabulated the results to show that the included studies did not provide strong evidence of the effectiveness of vitamin D supplementation in alleviating depression. The role of vitamin D in alleviating depressive symptoms or depression remains a subject of considerable debate within the scientific community.

Considering the potential for significant variation in vitamin D levels among population subgroups defined by factors such as age, gender, body mass index (BMI), and smoking status, it is important to account for these variables when assessing the relationship between vitamin D and health outcomes (23), the evaluation of the efficacy of vitamin D supplementation for depression in different populations should also be the subject of targeted studies. In recent years, the antidepressant effects of vitamin D in different populations remain controversial, and the biological mechanisms involved and other potential effects that may result from vitamin D interventions have not been clarified. Only one meta-study published in 2023 has systematically collated the effects of vitamin D on depression scores in older patients (24). However, given the limited number and evident heterogeneity of studies included in the previous meta-analysis, coupled with the emergence of new randomized controlled trials in recent years, we undertook a novel systematic review and meta-analysis. The aim was to assess the efficacy of vitamin D supplementation in alleviating depressive symptoms among older adults, in comparison with placebo. This analysis was conducted to provide guidance for clinical practice, with the results being specifically reflected in depression scale scores.

## 2 Information and methods

### 2.1 Registration and reporting

Literature search, screening, inclusion, and reporting were based on the guidelines of the Preferred Reporting Items for Systematic Reviews and Meta-Analyses 2020 (PRISMA 2020) (25). It was registered in the International Prospective Systematic Reviews Registry (PROSPERO) database (registration number CRD42024534071).

### 2.2 Search strategy

Two researchers (FJM and ZYC) independently conducted a systematic search of PubMed, EMBASE, Web of Science, and the Cochrane Library and covered all potentially relevant articles to assess the efficacy of vitamin D supplements for depression in the older. The timeframe of the literature search was limited from the creation of the databases to March 2024. Title- and abstract-based identification was performed using data available in each database. Full text was assessed only for studies defined as potentially eligible after a title- and abstract-based process. To obtain the full text of the studies, for those not available in the above databases and libraries, the corresponding authors were asked to provide it. All stages of appraisal were carried out independently by two researchers, but in the event of disagreement, a third researcher (YX) was asked to comment. The full search strategy for each database is shown in Supplementary material S1.

### 2.3 Study selection

Studies were considered eligible if they met the following criteria:

1. The included study was a randomized controlled trial;
2. Subjects were older adults aged >65 years with or without depressive symptoms or depression;
3. The control group did not receive vitamin D supplementation throughout the study, but only received the placebo study;
4. Subjects’ psychological status was assessed at baseline and at the end of the intervention using the Depression Assessment Scale based on their psychological symptoms.

The following studies were excluded:

1. Studies conducted in animal models;
2. Studies not published in English;
3. Studies that were only a research protocol or did not report results;
4. Interventions that included other nutritional supplements/drugs;
5. Studies for which the full text was not available or for which usable data could not be extracted;
6. Repeatedly published studies (the latest published or most complete study will be selected for inclusion).

### 2.4 Data extraction

Retrieved articles were independently screened by two authors and data were extracted according to a pre-designed data extraction form. Any disagreements were resolved through third-party discussions. Data extracted from each study included: authors; year of publication; country; duration of follow-up; participant characteristics (e.g., mean age, sex, sample size, 25(OH)D serum levels at baseline and endpoint); vitamin D dosing regimen followed; trial registration number; and means and standard deviations of participant depression assessment scale scores at baseline, endpoint, and differences between the two. The scales used to assess psychological symptoms were not uniform across studies, and when multiple depression assessment scales were used in the same study, preference was given to the primary outcome indicator from the original study or the better-known and more commonly used scale. Changes in pre- and post-scores were assessed by pooling standardized mean differences (SMDs) to find associations between vitamin D supplementation and improvements in depressive symptoms. Vitamin D dosage units were converted to international units per day (IU/day) to ensure consistency of data. Secondly, in addition to focusing on the primary outcome of depression, we extracted and documented data on secondary outcomes that may result from vitamin D in individuals in order to assess other benefits from vitamin D interventions.

### 2.5 Quality assessment and outcome measures

We assessed the quality of individual trials using the Cochrane Collaboration risk of bias tool, which summarizes the risk of bias for different items (26). These included information on randomized sequence generation (selection bias), allocation concealment (selection bias), blinding of subjects and researchers (performance bias), blinding of outcome assessors (detection bias), handling of incomplete data (lost to visit bias), and selective reporting of results as originally mentioned (reporting bias). After examining the full text of the included articles, the authors categorized the experimental risk as high, unclear, and low risk based on the above parameters.

The efficacy outcome metric for this meta-analysis was the difference in depression scores from baseline to endpoint between the experimental and control groups, which were assessed by several depression assessment scale. FJM and ZYC independently assessed the risk of bias, and any discrepancies were resolved through consultation with YX.

### 2.6 Statistical analyses

Vitamin D supplementation on depressive symptoms in older patients was assessed by combining each study of the total difference in change in depressive symptoms between the vitamin D group and the control group using Revman 5.3 software. For the whole sample, heterogeneity tests were estimated using the Cochrane chi-square test and inconsistency tests were used. A random effects model was applied to account for differences in the type and dose of vitamin D supplements. All probabilities (*p*-values) of the data were two-sided and *p* < 0.05 was considered statistically significant.

Potential variables contributing to sources of heterogeneity were investigated by subgroup analysis. Potential variables included: subjects’ baseline serum 25(OH)D levels, subject depression at baseline, dose of vitamin D supplementation, sex of subjects, duration of intervention, latitude of the study area and depression evaluation tool used for the study. Subjects were divided into vitamin D sufficiency group and vitamin D insufficiency or deficiency group by baseline vitamin D level of 30 ng/mL, vitamin D supplementation dose was divided into high-dose and low-dose groups by 2000 IU /day, intervention duration was divided into long-term and short-term intervention groups by 12 months, and latitude of the study area was divided into high-latitude and low-latitude by 45°. When ≥10 studies are included, publication bias will be assessed using funnel plots. In addition, the software is used for sensitivity analyses to estimate whether it affects the combined effect by excluding each study in turn.

## 3 Results

### 3.1 Study selection

**Figure 1:**
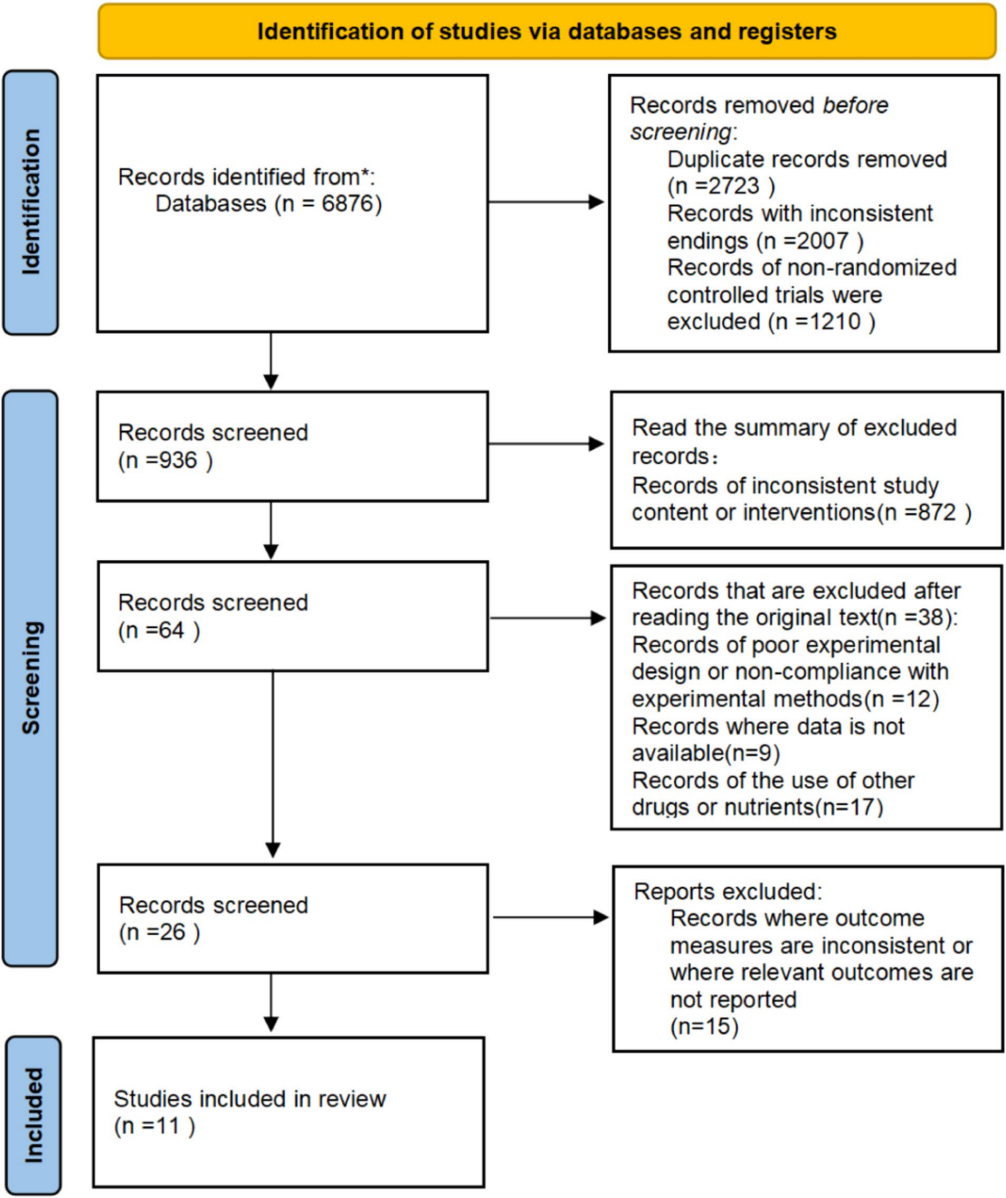
Flow chart of the study selection process.

A total of 6,876 relevant studies were evaluated after the database search. After removing 2,723 duplicate studies and 1,210 non-RCT studies, 910 studies were excluded by screening titles and abstracts. Of these, 872 study protocols were excluded due to study mismatch or intervention/control inconsistency, 12 studies were excluded due to poor experimental design or experimental methodology, 9 studies were excluded due to unavailability of data, and 17 studies were excluded due to supplementation with other drugs or nutrients. In addition, in the full-text evaluation and appraisal of the remaining 26 studies, 15 study protocols were excluded because the outcome metrics were inconsistent with the present study and because they did not report relevant results. Ultimately, 11 eligible studies were included based on the inclusion and exclusion criteria (27–37) (Figure 1).

### 3.2 Study characteristics

Table 1 synthesizes data from 11 randomized controlled trials conducted in different countries, covering participants from different countries and regions. Of these, three were conducted in Australia (28–30), two in the USA (27, 31), and the remainder were scattered across the Netherlands (34), Finland (32), Iran (33), Greece (35), the UK (36) and Turkey (37). The sample sizes of these studies varied significantly, ranging from a relatively small group of 77 individuals to a massive group of 16,822 individuals. In terms of treatment, the vast majority of studies used oral vitamin D3 (cholecalciferol), with only one study using both oral and intramuscular vitamin D supplementation for the intervention group. The daily dose of vitamin D3 varied widely, from 57 IU to as high as 30,000 IU. The duration of follow-up in the studies also varied, from a relatively short 4 weeks to as long as 5 years. Among the identified studies, scales used to assess depressive symptoms include the Patient Health Questionnaire (PHQ-9) (28, 35), the Positive and Negative Affect Scale (PANAS) (29), the Geriatric Depression Scale (GDS) (27, 31, 33), the Center for Epidemiological Studies Depression Scale (CES-D) (34), the World Health Organization Indicator of Wellbeing Scale (WHO-5) (30, 32), the 12-item Short Form Health Questionnaire (SF-12) (30, 36), and the 36-item Short Form Health Questionnaire SF-36 (34, 37). Out of the 11 studies, only three studies’ results supported a significant improvement of vitamin D on depression scores in older depressed patients, two of which (33, 34) included subjects with a confirmed diagnosis of depression at baseline, and the other study (35) included subjects with pre-diabetes and vitamin D insufficiency; the results of the remaining eight studies showed that vitamin D did not have a significant effect.

**Table 1:** Characteristics of included studies.

Author, year	Country	Sample size (experimental group/control group)	Vitamin D dose (IU/day)	Follow-up weeks (months)	Participant inclusion criteria characteristics (baseline characteristics)	Depression scale	Therapeutic evaluation
Vinod Yalamanchili et al., 2013	American (27)	246 (123/123)	0.25 g twice daily (200)	36	Older and postmenopausal women	GDS-LF30	Negative
Sabbir T. Rahman et al., 2023	Australian (28)	16,822 (8,552/8270)	60,000 IU per month (2000)	60	Potential participants between age 60 and 79 years	PHQ-9	Negative
Ian T. Zajac et al., 2020	Australian (29)	181 (89/92)	(600)	12	Healthy men and women aged between 60 and 90 years old; Fluent in English (effective cognitive test completion); Have not take any form of vitamin D supplement for at least three months before this study and are willing to avoid additional vitamin D supplementation for the duration of this study	PANAS; DASS−21; GHS	Negative
Kerrie M. Sanders et al., 2011	Australian (30)	2012 (1,001/1011)	500,000 IU / year, 10 tablets oral (1370)	36–60	Older women (70 years old) living in the community	GHQ, SF-12, WHO-5	Negative
Vinod Yalamanchili et al., 2018	American (31)	273 (238/35)	Take vitamin D seven times daily, 400 IU (400–4,800)	12	Older Caucasian and African-American older women; Serum 25 OHD 20 ng/mL (50 nmol / L); Age between 57–90 years	GDS-LF30	Negative
Radhika Patil et al., 2016	Finnish (32)	183 (88/95)	(800)	24	Community, healthy, older women with adequate vitamin D levels	WHO-5	Negative
Negin Masoudi Alavi et al., 2018	Iran (33)	78 (39/39)	50,000 IU per month (7,143)	2	Over 60 years old; Iranian nationality, who can answer questions in Persian for medical treatment; Moderate to major depressive disorder (GDS > 5 points)	GDS-15	Positive
Elisa J de Koning, et al., 2019	The Netherlands (Amsterdam) (34)	153 (75/76)	(1200)	12	presence of depressive symptoms; (CES-D) Score of 16; 25 (OH) D concentration at 15 to 50 nmol / L	CES-D, BAI, SF-36	Positive
Evangelia Zaromytidou et al., 2022	Greek (35)	77 (42/35)	(2000)	12	More than 60 years old; Pre-diabetes; The 25 (OH) D level is below 30 ng/mL; There was no history of diabetes, nephropathy, cancer, inflammatory, rheumatic, or psychiatric disorders	STAI, PHQ-9	Negative
J. C. Dumville et al., 2006	British (36)	1,621 (680/941)	(3800)	6	Female, aged 70 years or older; Participate in the trial from May to October; The MCS score of the SF-12 questionnaire at baseline was valid	SF-12 (MCS)	Negative
Hakan Sakalli et al., 2012	Turkish (37)	120 (60/60)	Oral and parenteral route, single high dose (300000)	1	Community older subjects aged over 65 years; Patients over 65 years old who went to the outpatient department of Rheumatology department of our hospital for various reasons; Cases with vitamin D treatment were excluded	SF-36	Negative

CES-D, Center for Epidemiological Studies Depression Scale; PHQ-9, Patient Health Questionnaire-9; PANAS, Positive and Negative Affect Schedule; DASS-21, The Depression Anxiety Stress Scale 21; GHS, the General Happiness Scale; GHQ, General Health Questionnaire; SF-12, Short Form 12 Health Survey; WHO-5, The World Health Organization- Five Well-Being Index; GDS-LF30, The 30-item Geriatric Depression Scale, GDS-15, Geriatric Depression Scale-15; STAI, State–Trait Anxiety Inventory; MCS, Mental Component Summary; SF-36, 36-Item Short Form Survey.

The baseline characteristics of the study participants are presented in Table 2. The study recruited people whose mean age ranged from 66.3 to 76.8 years. There were five research (27, 30–32, 36) where the experimental subjects were exclusively female, indicating that a large percentage of female subjects comprised the gender distribution. Moreover, patients in six trials (31–35, 37) had vitamin D insufficiency or deficiency at baseline, and the mean vitamin D levels in these studies ranged from 17.6 ng/mL to 31.7 ng/mL. Regarding the participants’ health, the subjects of three studies were patients with depression, three additional research focused on healthy people, and the subjects of the remaining five studies were not specified.

**Table 2:** Study population baseline information.

First author, year	Age (mean ± SD)		Female proportion (%)		Vitamin D level (ng/mL)		Depression (%)	
	Vitamin D group	Placebo group	Vitamin D group	Placebo group	Vitamin D group	Placebo group	Vitamin D group	Placebo group
Vinod Yalamanchili, 2013	71.8 ± 3.4	71.1 ± 3.7	100	100	30.6 ± 9.4	31.7 ± 11.0	9.8^a^	13.8^a^
Sabbir T. Rahman, 2023	69.3 (60–84)	69.3 (60–84)	46	46	NA	NA	NA	NA
Ian T. Zajac, 2020	70.8 ± 6.4	70.1 ± 5.7	53.8	51.1	31.1 ± 0.8	29.7 ± 0.8	0	0
Kerrie M. Sanders, 2011	75.8 (72.9–79.9)	75.8 (72.9–79.2)	100	100	NA	NA	NA	NA
Vinod Yalamanchili, 2018	66.3, 66.6 (57–90)	66.3, 66.6 (57–90)	100	100	NA	NA	NA	NA
Radhika, Patil 2016	74.1 ± 3.0	73.8 ± 4.1	100	100	26.28 ± 6.92	27.44 ± 7.36	NA	NA
Negin Masoudi Alavi, 2018	68.7 ± 7.0	67.0 ± 6.3	48.7	51.3	22.57 ± 6.2	21.2 ± 5.8	100^b^	100^b^
Elisa J. de Koning, 2019	67.8 (65.4–71.7)	67.3 (63.4–72.0)	58.4	56.4	18.4 (13–22.8)	17.6 (14.4–22.1)	100^c^	100^c^
Evangelia Zaromytidou, 2022	73.1 ± 7.2	74.0 ± 7.6	80	77.8	19.98 ± 6.73	19.85 ± 5.72	NA	NA
J. C. Dumville, 2006	77.0 ± 5.1	76.7 ± 4.9	100	100	NA	NA	NA	NA
Hakan Sakalli, 2012	69.8 ± 3.7	68.9 ± 2.7	46.6	50	20.9 ± 9.5	21.2 ± 7.4	NA	NA

^a^GDS-LF30, Geriatric Depression Scale Long Form 30 (0 ~ 10 = normal, 11 ~ 30 = depressed). ^b^GDS-15, Geriatric Depression Scale 15 (0 ~ 4 = normal, 5 ~ 15 = depression). ^c^CES-D, Center for Epidemiological Studies Depression Scale (0 ~ 15 = normal, 16 ~ 60 = depression).

### 3.3 Assessing the quality of evidence across studies

**Figure 2:**
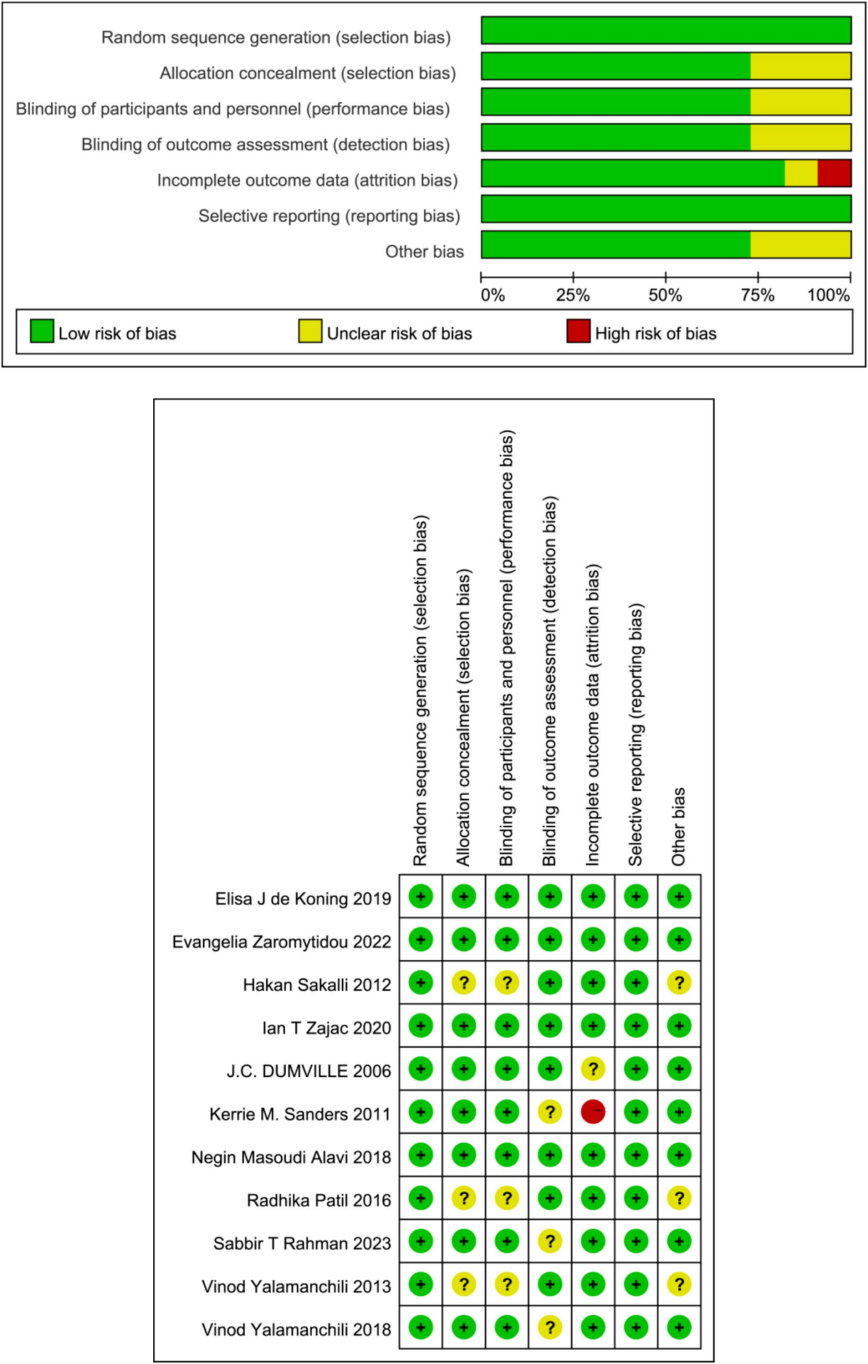
Quality assessment of final screening studies.

The quality of evidence obtained from the included trials was assessed as low risk, but there was always a risk of unexplained heterogeneity and selective reporting of outcome bias. Six trials (27, 28, 30–32, 36, 37) may have been at risk of blinding bias and one RCT was at significant risk of bias (30) (Figure 2).

### 3.4 The results of the meta-analysis

#### 3.4.1 General impact of vitamin D supplementation on older persons’ depression therapy

**Figure 3:**
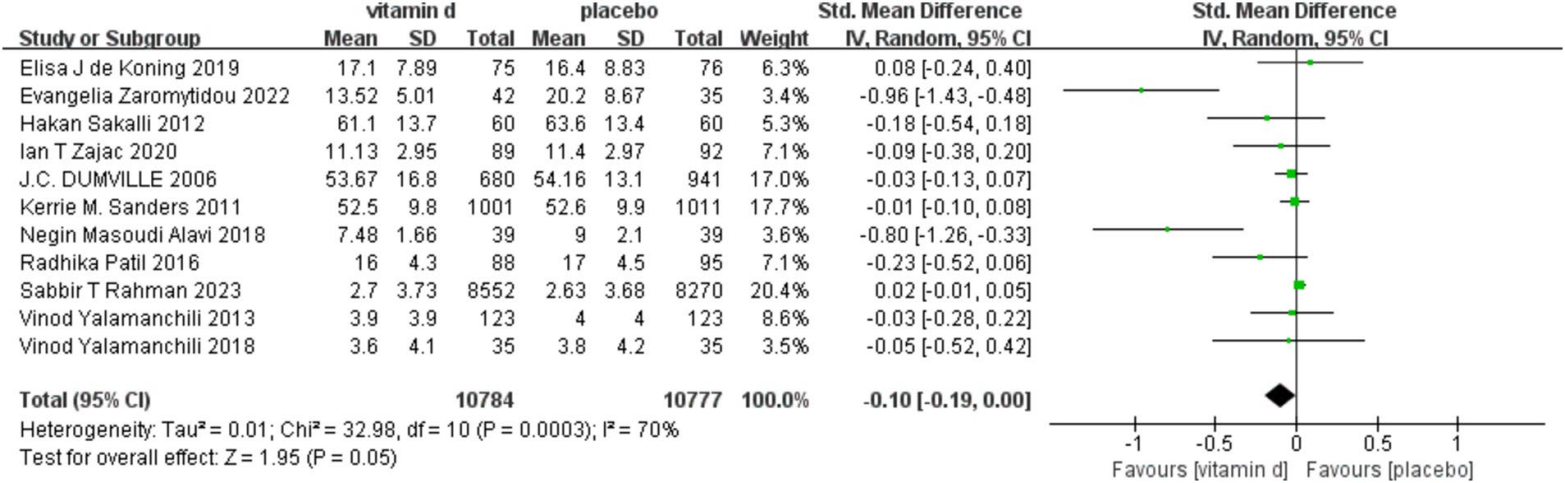
Forest plot of correlation between vitamin D supplementation intervention and depression scale scores compared with placebo.

Because the depression rating scale used in the included studies was not uniform, we used a random-effect model. Four studies (29, 30, 34, 35) used multiple scales simultaneously to assess depressive symptoms, and we extracted scores for the primary outcome indicators. For the mean and SD of post-intervention scores for depressive symptoms, one study (33) obtained them from graphical and two studies (34, 36) obtained them by calculation. Combining data from 11 studies, a pooled analysis revealed (Figure 3) that vitamin D supplementation did not significantly affect depression [SMD: −0.10; 95% CI: (−1.19, 0.00); *p* = 0.05]. However, there was a decrease in depression assessment scale scores from baseline to endpoint in the experimental group when compared to the control group, indicating that supplementation may have some overall effect on treating depression in the older. There was also a lot of study heterogeneity (I^2^ = 70%; χ^2^ = 32.98; *p* < 0.001) (Figure 3).

#### 3.4.2 Subgroup analyses

**Figure 4:**
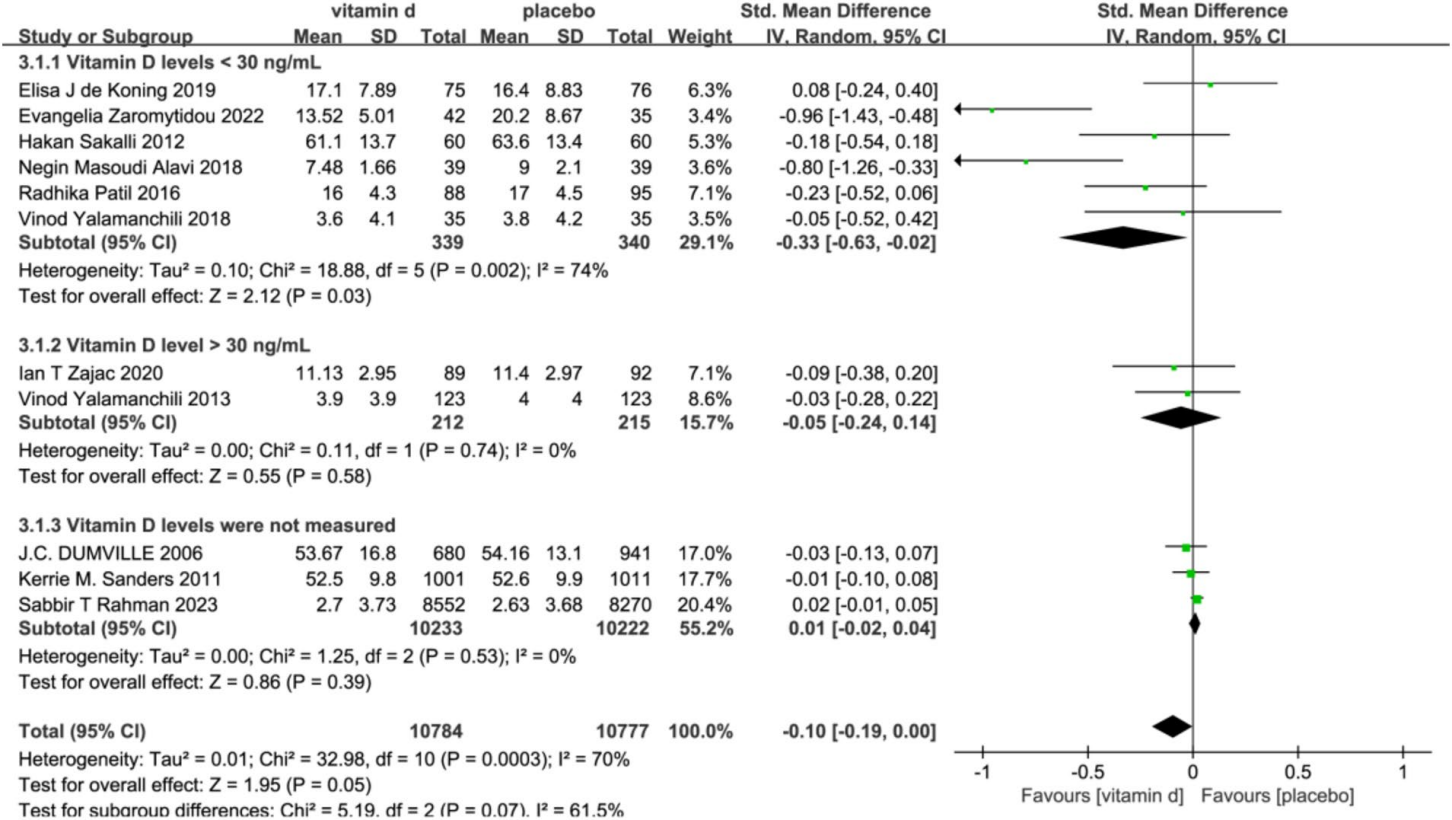
Forest plot of correlation between vitamin D intervention and geriatric depression scale score in a population with different baseline vitamin D levels.

**Figure 5:**
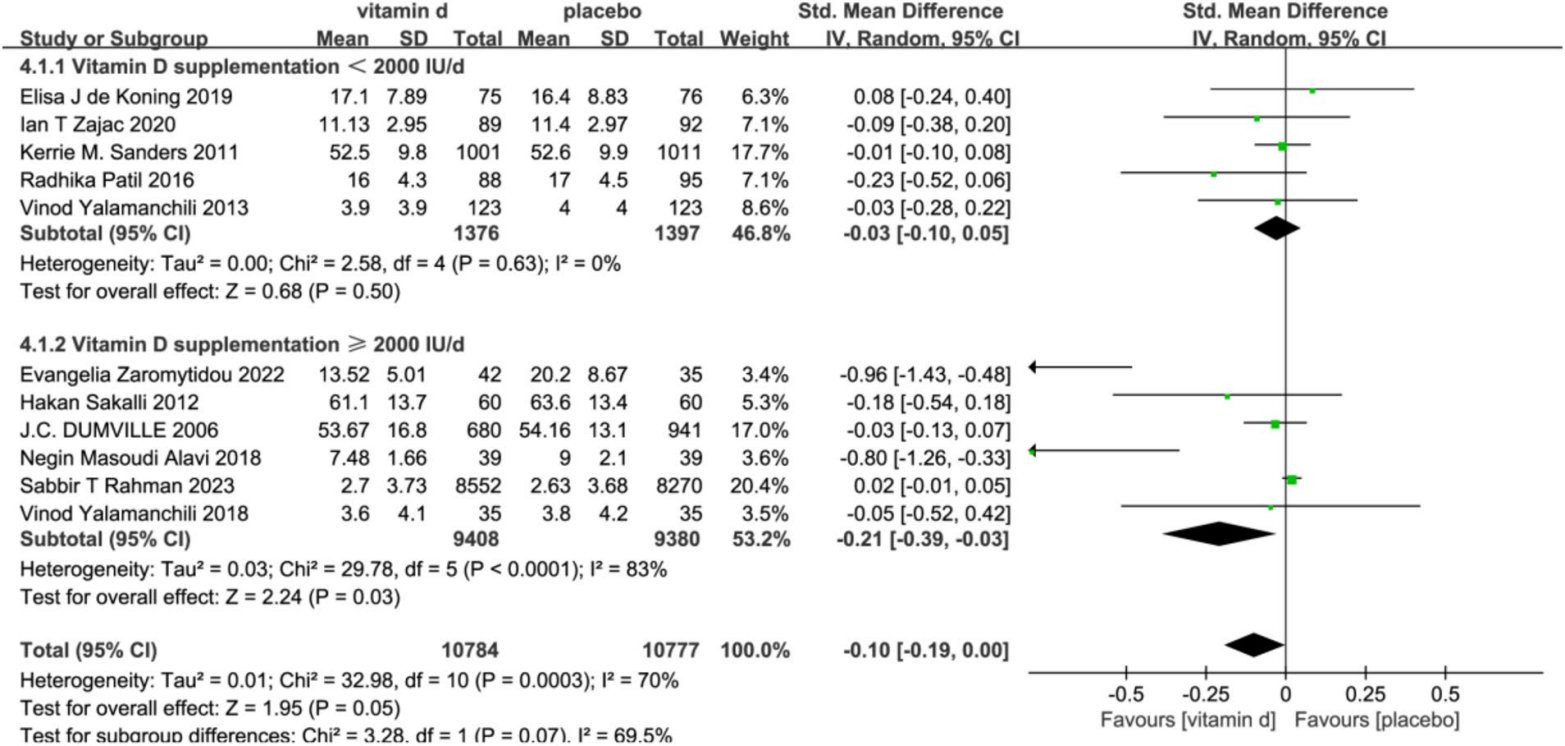
Forest plot of correlation between different doses of vitamin D supplementation. Interventions and depression scale scores.

In the subgroup analyses, six studies with 679 subjects with baseline serum 25(OH)D levels <30 ng/L were classified as vitamin D insufficient or deficient. The vitamin D intervention had a significant effect on reducing scores on the Geriatric Depression Scale compared with the placebo group [SMD: −0.33; 95%CI: (−0.63, −0.02); *p* = 0.03]. In contrast, five studies included subjects with serum 25(OH)D levels ≥30 ng/L or unmeasured at baseline, and the intervention effect of vitamin D was not significant (Figure 4). To assess the differences in effectiveness of vitamin D supplementation by dosage, 11 studies were analyzed by dividing the 11 studies into a low-dose group (<2000 IU/day) and a high-dose group (≥2000 IU/day) in five and six studies, respectively. The combined effect (SMD) of low and high-dose vitamin D supplementation was −0.03 and −0.21, respectively. The results showed that high-dose vitamin D supplementation may be effective in alleviating depressive symptoms and treating depression in older adults, while low-dose vitamin D supplementation is futile (Figure 5).

The duration of the vitamin D supplementation intervention may also have some effect on depression scores in the older. Using 12 months as a cut-off, seven of the included studies had a follow-up period of less than 12 months, and only four had a longer period. However the combined effect of shorter interventions [SMD: −0.07; 95%CI: (−0.18, 0.05); *p* = 0.26] and longer interventions [SMD: −0.06; 95%CI: (−0.14, 0.03); *p* = 0.21] was not significantly different when compared to the placebo group, and does not support an effect of the duration of vitamin D interventions on the depressive outcome.5 studies, which included only female subjects, the vast majority of the 2,588 women being menopausal, showed that the vitamin D intervention was effective in reducing depression scores compared to placebo [SMD: −0.19; 95%CI: (−0.44, 0.05); *p* = 0.11], whereas this effect was not demonstrated in the other six studies, which had a more balanced gender ratio (SMD: 0.01). Three studies included patients with depressive symptoms or diagnosed depression at baseline, although the sample size was only 475, and for this subject, the vitamin D intervention was also found to contribute to the effect of depressed mood [SMD: −0.21; 95%CI: (−0.65, 0.23); *p* = 0.35]. The study in the regional latitude was related to vitamin D supplementation, with 8 studies conducted in lower latitude areas and three in higher latitudes, results of vitamin D [SMD: −0.14; 95%CI: (−0.27, 0.00); *p* = 0.04] depression intervention than higher latitude [SMD: −0.04; 95%CI: (−0.15, 0.06); *p* = 0.40] 0.11 studies used six different assessment scales for depression assessment, and subgroup analyses showed no significant differences in the effects of the vitamin D intervention evaluated by the different assessment scales (Table 3).

**Table 3:** Subgroup analysis of the correlation of vitamin D supplementation on depression scores in older adults.

	Number of studies (sample size)	Pooled SMD (95% confidence interval)	I^2^ (%)	The *p*-value (one group difference)
Baseline serum vitamin D level				
Vitamin D insufficiency or deficiency (<30 ng/mL)	6 (679)	−0.33 [−0.63, −0.02]	74	0.03
Vitamin D was adequate (> 30 ng/mL)	2 (427)	−0.05 [−0.24, 0.14]	0	0.58
Vitamin D levels were not tested	3 (20455)	0.01 [−0.02, 0.04]	0	0.39
Intervention to supplement the vitamin D level				
<2000 IU/d	5 (2773)	−0.03 [−0.10, 0.05]	0	0.5
≥2000 IU/d	6 (18788)	−0.21 [−0.39, −0.03]	83	0.03
The duration of follow-up				
Less than 12 Months	7 (19561)	−0.07 [−0.18, 0.05]	69	0.26
12 Months of disease	4 (2042)	−0.06 [−0.14, 0.03]	0	0.21
Sex				
The study was limited to women only	5 (2588)	−0.19 [−0.44, 0.05]	75	0.11
Study gender balance	6 (18973)	0.01 [−0.02, 0.04]	66	0.51
Baseline depression	3 (475)	−0.21 [−0.65, 0.23]	81	0.35
Baseline health, with no confirmed disease	7 (21135)	0.01 [−0.02, 0.04]	0	0.54
Latitude				
High-latitude	3 (1955)	−0.04 (−0.15, 0.06)	7	0.40
Low-latitude	8 (19606)	−0.14 (−0.27, 0.00)	76	0.04
Depression scale				
PHQ-9	2 (16899)	−0.44 [−1.39, 0.52]	94	0.37
PANAS	1 (181)	−0.09 [−0.38, 0.20]	NA	0.54
GDS	3 (394)	−0.27 [−0.73, 0.20]	77	0.26
CES-D	1 (151)	0.08 [−0.24, 0.40]	NA	0.61
WHO-5	1 (183)	−0.23 [−0.52, 0.06]	NA	0.13
SF-12, SF-36	3 (3753)	−0.03 [−0.09, 0.04]	0	0.44
Sensibility analysis				
High risk studies excluded	10 (2258)	−0.14 [−0.26, −0.01]	73	0.03
Studies with shorter duration (<6 months)	7 (19561)	−0.07 [−0.18, 0.05]	69	0.26
Fixed effect model	11 (21561)	0.00 [−0.02, 0.03]	70	0.88

### 3.5 Secondary outcomes

#### 3.5.1 Quality of life

A total of three studies (30, 32, 37) scored quality of life in older adults before and after vitamin D supplementation. The SF-12, Leipad questionnaire, and SF-36 were used. All three studies showed no significant association between vitamin D supplementation and quality of life, although the intervention group supplemented with vitamin D may have shown higher quality of life scores and a more positive convergence. In addition, one study (36), although using the SF-12, assessed only the psychological component and could not directly account for quality of life.

#### 3.5.2 Fear of falling

Only one study (32) assessed fear of falling using 2 methods, the Falls Efficacy Scale International (FES-I) and the Visual Analogue Scale (VAS). The former focused on quantifying the differentiation between categories of fear of falling and the latter on overall fear of falling. Results showed a statistically significant difference in FES-I scores compared to controls after a one-year vitamin D intervention, while VAS scores did not improve.

#### 3.5.3 Inflammatory markers

One study (35) measured white blood cells (WBC) before and after a vitamin D intervention. Following a 12-month intervention period, there was a significant increase in WBC levels in the control group as compared to baseline levels. However, this increase was not observed in the intervention group (Figure 5).

### 3.6 Publication bias and sensitivity analyses

**Figure 6:**
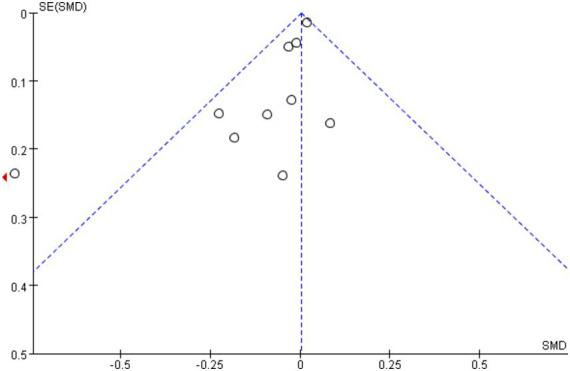
Funnel plot of the effect of vitamin D on depression scores.

To assess publication bias, funnel plots of improvement in depressive symptoms with vitamin D were derived. The funnel plots in our results all appeared to be symmetric with the effect estimates, suggesting that there was no significant publication bias in our study (Figure 6). Nevertheless, when Egger’s test was run, the results indicated *p* = 0.0001 < 0.05, indicating that the study contained some publication bias. Next, we used cut-and-patch to confirm whether the publication bias between the studies had an impact on the analysis’s findings, and the results indicated that the study’s findings were robust and that the significance of the effect values had not changed significantly. Finally, we employed meta-regression to investigate additional possible sources of heterogeneity, and all of the included variables showed *p* > 0.05, indicating that no significant sources of heterogeneity had been found.

We also performed sensitivity analyses, and by excluding studies with a high risk of bias and short intervention duration, and applying a fixed-effects model, the results support the stability of our results, with no statistically significant effect of vitamin D supplementation on depression. Additionally, we assessed the findings’ sensitivity to each included study by eliminating them one at a time; the sensitivity analysis graph is displayed in the Appendix. We found no affect on the stability of our results.

## 4 Discussion

This systematic review encompassed previous randomized controlled trials to assess the impact of vitamin D supplementation on depressive symptoms in older patients, resulting in a comprehensive analysis of 11 trials. The meta-analysis indicated no significant association between vitamin D supplementation and the amelioration of depression in this population [SMD: −0.10; 95% CI: (−1.19, 0.00); *p* = 0.05]. Given the substantial heterogeneity observed among the studies, we conducted stratified analyses based on several factors, including baseline serum 25(OH)D levels, baseline depression diagnosis, gender, intervention dosage, intervention duration, geographic latitude, and the depression assessment scale. Subgroup analyses revealed that vitamin D supplementation was more effective in improving depressive symptoms for individuals with baseline depressive symptoms or depression, those with vitamin D insufficiency, residents of low-latitude regions, and women. Furthermore, the efficacy of vitamin D intervention was found to correlate with the dosage and duration of supplementation. However, the results were still not significant.

Vitamin D did not significantly reduce depression, according to the results of either our study or the systematic review published by Park in 2023 (24), which both focused on the aged population and had comparable study designs and contents. The age definition and retrieval procedures of the older do not change, though, and we have conducted a more thorough study and discussion of the secondary outcomes of vitamin D intervention, as well as any mixed variables that could be present during the intervention process. Secondly, this systematic study contradict some previous meta-analyses of vitamin D supplementation for the prevention and treatment of depression conducted in other populations. The difference between the results of the before and after studies is closely related to the inclusion of the characteristics of the study population, the intervention strategy, and the analysis of the outcomes in each study. Furthermore, the potential for publication bias across meta-analyses may significantly influence the outcomes. It is noteworthy that previous meta-analytical findings suggest that vitamin D supplementation may exert a more pronounced effect on depressive symptoms in younger and psychologically healthier populations. Vitamin D may only have a preventative effect on depression and no therapeutic effect on already diagnosed depression or more severe depressive symptoms. If this argument is valid, the subjects included in the study should have been assessed for psychological status at baseline and grouped into subgroups, and it would be an unreasonable experimental design to mix the presence/absence of depression for the intervention analyses. There is also a paucity of data supporting vitamin D’s therapeutic impact on various levels of depression. Only two of the included studies were totally diagnosed with depression, and there is a lack of more extensive analysis, which will need to be confirmed by future research.

When we investigated causes of heterogeneity using subgroup analyses and meta-regression, we found no influence of any subgroup on the stability of the results. We are, however, worried about the potential issues provided by the employment of several assessment measures. Because of the disparities in objectivity and accuracy across different scales, it is difficult to assure the stability and consistency of our experimental data, and it highlights the influence of multiple measuring methods. Second, there is no proof that vitamin D supplements usually reduce depression symptoms in patients with various health issues. Baseline health problems, comorbidities, and medication usage all have some effect on vitamin D’s role in the body. Comorbidities such as liver disease, cardiovascular disease, and neurodegenerative disease, for example, will affect the twice hydroxylation of vitamin D in the body, affecting its bioavailability (38); medication use may affect the activity of vitamin D-related enzymes, and vitamin D, in turn, may alter drug action and disposition in the body (39). Even though our subgroup analyses indicate that the findings are robust and insensitive, many possible sources of heterogeneity must be investigated further.

The extra-skeletal effects of vitamin D have not been established, and the plausibility of its association with depression has not yet been demonstrated, although the results of the present study do not support a significant therapeutic effect of vitamin D on depression, based on previous experimental studies of the association, it is important to consider the potential mechanisms by which vitamin D may affect depression. Vitamin D receptors are widely present in brain regions associated with depression, such as the amygdala, substantia nigra, and hippocampus (40, 41) and vitamin D crosses the blood–brain barrier, activates vitamin D receptors, and plays a role in the control of behavior in humans (42), and its deficiency has been associated with a reduction in brain tissue and hippocampal volume in animals or humans (43). In addition, vitamin D regulates the release of neurotransmitters and the synthesis of brain-derived neurotrophic factors (44), and it regulates the release of monoamine neurotransmitters such as epinephrine, norepinephrine, and dopamine via vitamin D receptors in the adrenal cortex (45) as well as preventing their depletion (46), and thus plays a role in mood-, reward-, and anxiety-related behaviors. Vitamin D deficiency also affects GABA-A receptor activity below a certain level, which in turn affects HPA axis disorders (e.g., hyperactivity or negative feedback dysfunction), resulting in disorders of the organism’s stress system (47, 48). Vitamin D serves several activities in the brain, including neuroimmune regulation, neurotrophic factor modulation, neuroprotection, and neuroplasticity (49). Vitamin D’s preventive effect on depression may be based on its protective effect on brain nerves. Vitamin D has an antioxidant effect in the central nervous system, enhances gene expression of nerve growth factor and antioxidants, and downregulates cytokines and inflammatory mediators, such as nuclear factor-kB (50). Furthermore, vitamin D has been demonstrated to lower plasma C-reactive proteins in individuals with mental illnesses and control inflammation (51) by suppressing pro-inflammatory cytokines. Long-term vitamin D supplementation can improve cognitive ability (52), immune function, and musculoskeletal condition (53), which may indirectly affect the participants’ mental health and, to some extent, may be a protective factor of depression. The effects of vitamin D on mental health are currently largely limited to depression, and there is also a lack of clear evidence confirming its mechanism of action; more basic experimental studies are needed to investigate this in the future.

Differences in the effectiveness of vitamin D supplementation for depression treatment may stem from the absence of standardized clinical thresholds for vitamin D in treatment protocols. The current uncertainty regarding the optimal serum levels of vitamin D and the inconsistency in recommended supplementation regimens underscore the necessity for population-specific supplementation guidelines. In formulating recommendations aimed at alleviating depressive symptoms, critical factors include the dosage of supplementation, its duration, the method of administration, and potential synergies with other therapeutic interventions. Our analysis indicates that higher doses of vitamin D, exceeding 2000 IU per day, may be more effective for preventing and treating depression, in contrast to lower doses that do not surpass this threshold, which are deemed less efficacious. The results should also be interpreted with caution due to the large disparity in the doses of the included experimental interventions and the presence of monthly or annual dosing in four studies (28, 30, 33, 37). There is no accurate cut-off value for the threshold dose of vitamin D intervention on depressive symptoms. It is hoped that future larger population-targeted cohort studies could fill this gap. Our subgroup analysis of baseline serum 25(OH)D showed that the depressive efficacy of vitamin D supplementation was more effective in those with insufficient or deficient serum 25(OH)D. However, only a small proportion of the 11 studies included in this meta-analysis had serum 25(OH)D testing, and large cohort studies did not observe this metric (28, 30, 36). Furthermore, the human body uses serum active vitamin D (1,25 hydroxyvitamin D), which is a byproduct of two hydroxylations of vitamin D, and it is a more reliable measure of vitamin D levels in the body (54). Only one of the studies we included (31) examined active vitamin D concentrations in participants, and it would be more promising for future studies to focus more on serum active vitamin D concentrations. The safety of long-term administration of high doses of vitamin D needs to be considered, especially in the older. For the dose to be taken, the Endocrine Society’s clinical guidelines state that adults may need to consume 1,500–2,000 IU/day of vitamin D if they want to consistently increase their blood levels of 25OHD above 30 ng/mL (12). 4,000 IU/day is the upper limit for those aged 19–70 years, and 10,000 IU/day is the upper limit for those aged 70 years or older above that level. Long-term use may increase the likelihood of adverse effects such as hypercalcemia and renal calcium deposits. Further large studies are still needed in the future to validate the potential benefits and safety of vitamin D in the clinic. For the duration of intervention, a longer period is needed to observe the body’s response to vitamin D supplementation because vitamin D has to work by binding to the vitamin D receptor and affecting transcription in the nucleus, inducing value-added and differentiation of cells of different lineages. In contrast, the follow-up period of the studies included in this meta-analysis was longer overall, and subgroup analyses with a 12-month cut-off did not reveal large differences in the results, with both subgroups showing some improvement in depressive symptoms by vitamin D. There is also no clear conclusion about the possible time point for triggering the vitamin D organism response. It has been suggested that vitamin D interventions of more than 2 months can have a more significant improvement in depressive symptoms (18), and only one of the studies included in this meta-analysis had an intervention duration of less than 2 months (37), which may explain why the treatment effects of the two subgroups stratified by the duration of the intervention in the present study were not significantly different. In addition, although many RCT trials are interested in supporting the antidepressant effect of vitamin D, the present study did not have sufficient evidence to support the efficacy of vitamin D supplementation on depressive symptoms; therefore, we conclude that vitamin D supplementation as a standalone intervention for preventing or treating depressive symptoms in older patients is not sufficiently reliable. It is essential that dietary interventions be more diverse and holistic. For instance, we advocate for dietary structures that are more likely to enhance the alleviation of depression (e.g., the Mediterranean diet (55)), increasing the duration of sunlight, and the intensity of outdoor activities. Some studies have also suggested that food sources of vitamin D may be more effective than pills (56), and dietary intake high in vitamin D is more recommended than pill intake. The treatment protocol advocates for the integration of dietary interventions with complementary therapeutic strategies, including pharmacological treatment, psychotherapy, and physical exercise, to attain a more efficacious management of depressive disorders.

## 5 Limitations and strengths

The present investigation has certain evident analytical constraints, which are manifested in the following principal areas. First, it was challenging for us to determine with precision if vitamin D supplementation produced a significant difference between healthy older persons and those with depression because of the small sample size of the particular cohort with diagnosed depression. This restriction limited our ability to fully comprehend the effects of supplementation in both groups and the amount of vitamin D required. Second, even after subgroup analysis, the systematic evaluation retained a substantial degree of study heterogeneity, and we still need to consider other possible sources of heterogeneity. The systematic evaluation’s studies were carried out in various populations, and it is crucial to note that a number of variable factors pertaining to the subjects—such as their lifestyle, ethnicity, dietary habits, BMI, smoking status, amount of daily sunlight exposure, activity of specific enzymes related to vitamin D absorption, body’s natural capacity to synthesize vitamin D through sunlight exposure, physical activity, and adherence to supplementation—may have a substantial impact on the experiment’s outcomes. The intricacy and variety of these variable elements made it more challenging to appropriately interpret the outcomes of our experiments. Furthermore, we should not undervalue the difficulties presented by the variety of study designs. Variations in the vitamin D supplementation regimens (doses, intervals, durations of interventions, etc.) and depression assessment scales used by the studies could have biased our interpretation of the effects of vitamin D supplementation on depression and health, and as a result, the results might not have been comparable. Finally, the interaction between medications taken and vitamin D in the included study populations who had other underlying medical conditions is also impossible to estimate. Two studies in depressed populations, one study in a pre-diabetic population, and one study in a rheumatic population were included in this meta-analysis. Regarding the depressed population, there might be possible interactions between vitamin D and traditional depressants on depression symptoms, but it’s unknown if other drugs have any effect. On the other hand, a variety of other elements, including social relationships, life stress, and quality of life, also have an impact on the psychological well-being of the aged (3). This comprehensive study is biased since it only examines how vitamin D supplementation affects depression symptoms. In summary, although our study has made some progress in exploring the relationship between vitamin D and health and depression, the limitations mentioned above need to be overcome and improved in our future studies.

In this study, we conducted a thorough and contemporary review of randomized controlled trials that investigated the use of vitamin D supplementation for the alleviation of depressive symptoms. Our analysis integrated data from a broad spectrum of existing studies. The findings not only corroborate the effects noted in prior meta-analyses but also extend these validations through an examination of a more extensive and contemporary dataset. This approach helps to reconcile discrepancies present in earlier research. The robustness of our review and meta-analysis is primarily attributable to the inclusion of a diverse array of studies and a stringent assessment of methodological quality.

Further research is required to more precisely evaluate the efficacy of vitamin D in alleviating depressive symptoms among older adults. Future randomized controlled trials should employ standardized protocols for vitamin D dosing, utilize validated scales for assessing depression, and consider variables such as baseline serum vitamin D concentrations. These methodological enhancements will facilitate the establishment of robust evidence supporting the impact of vitamin D on depressive symptoms. Such rigorous studies will enhance our understanding of vitamin D’s role in the mental health of older individuals and inform the scientific basis for its potential therapeutic applications in depression treatment.

## 6 Conclusion

In aggregate, the present study does not definitively establish a direct correlation between vitamin D supplementation and marked amelioration of depressive symptoms among older adults. Nonetheless, our subgroup analyses indicate that high-dose, extended-duration vitamin D interventions may yield favorable antidepressant effects in older individuals with pre-existing vitamin D insufficiency or deficiency. Additionally, there is a suggestion that vitamin D could potentially ameliorate depressive symptoms in certain demographic groups. However, the extent and clinical significance of this effect remain to be conclusively determined.

## Data Availability

The original contributions presented in the study are included in the article/Supplementary material, further inquiries can be directed to the corresponding author.
