# Supplementary material for: Efficacy of vitamin D supplementation on depressive symptoms in older patients: a meta-analysis of randomized controlled trials
Source: Front Med (Lausanne). 2024 Oct 10;11:1467234. doi: 10.3389/fmed.2024.1467234 (PMC11500197; doi:10.3389/fmed.2024.1467234)

**Table S1.** Search strategies for the databases.

| **Database**  **(Search date)** | **Search strategy** | **Number of results** |
| --- | --- | --- |
| PubMed | depression[T/A] OR depressive disorder[T/A] OR depressive disorders[T/A] OR suicidal[T/A] OR suicide[T/A] OR anxiety[T/A] OR well-being[T/A] OR wellbeing[T/A] OR negative emotion[T/A] OR quality of life[T/A] OR self esteem[T/A] OR self-esteem[T/A] OR mental health[T/A] OR mental disorders[T/A] OR mental disorder[T/A] OR psychological distress[T/A] OR self efficacy[T/A] OR self-efficacy[T/A] OR resilience[T/A] OR empowerment[T/A] OR anxiety depression[T/A] OR depression anxiety[T/A] OR mood disorders[T/A] OR depressed individualsOR life skills[T/A] OR anhedonia[T/A] OR resilience[T/A] OR emotional[T/A] OR psychology[T/A] OR loss of interest[T/A] OR reduced energy socialOR participation[T/A] OR psychological distress[T/A] OR mental capital[T/A] OR restless[T/A] OR psychosocial[T/A] OR psychiatry[T/A] | 1,720,548 |
|  | vitamin D[T/A] OR vitamin D2[T/A] OR vitamin D3[T/A] OR D2[T/A] OR D3[T/A] OR ergocalciferol[T/A] OR cholecalciferol[T/A] OR 25(OH)D[T/A] OR 25-hydroxyvitamin D[T/A] OR 3-epi-25hydroxyvitaminDOR calcitriol[T/A] OR dihydroxycholecalciferol[T/A] | 165,901 |
|  | (((RCT[T/A]) OR (randomized controlled trial[T/A])) OR (randomized controlled[T/A])) OR (placebo[T/A])) OR (placebos[T/A]) | 509,785 |
|  | 1# AND #2 AND #3 | 266 |
| WOS | TS=depression OR depressive disorder OR depressive disorders OR suicidal OR suicide OR anxiety OR well-being OR wellbeing OR negative emotion OR quality of life OR self esteem OR self-esteem OR mental health OR mental disorders OR mental disorder OR psychological distress OR self efficacy OR self-efficacy OR resilience OR empowerment OR anxiety depression OR depression anxiety OR mood disorders OR depressed individualsOR life skills OR anhedonia OR resilience OR emotional OR psychology OR loss of interest OR reduced energy socialOR participation OR psychological distress OR mental capital OR restless OR psychosocial OR psychiatry | 1,419,035 |
|  | TS=vitamin D OR vitamin D2 OR vitamin D3 OR D2 OR D3 OR ergocalciferol OR cholecalciferol OR 25(OH)D OR 25-hydroxyvitamin D OR 3-epi-25hydroxyvitaminDOR calcitriol OR dihydroxycholecalciferol | 121,025 |
|  | TS=RCT OR randomized controlled trial OR randomized controlled OR placebo OR placebos | 514,625 |
|  | 1# AND #2 AND #3 | 1220 |
| Embase | depression OR depressive disorder OR depressive disorders OR suicidal OR suicide OR anxiety OR well-being OR wellbeing OR negative emotion OR quality of life OR self esteem OR self-esteem OR mental health OR mental disorders OR mental disorder OR psychological distress OR self efficacy OR self-efficacy OR resilience OR empowerment OR anxiety depression OR depression anxiety OR mood disorders OR depressed individualsOR life skills OR anhedonia OR resilience OR emotional OR psychology OR loss of interest OR reduced energy socialOR participation OR psychological distress OR mental capital OR restless OR psychosocial OR psychiatry | 420,963 |
|  | vitamin D OR vitamin D2 OR vitamin D3 OR D2 OR D3 OR ergocalciferol OR cholecalciferol OR 25(OH)D OR 25-hydroxyvitamin D OR 3-epi-25hydroxyvitaminDOR calcitriol OR dihydroxycholecalciferol | 143,405 |
|  | RCT OR randomized controlled trial OR randomized controlled OR placebo OR placebos | 668,743 |
|  | 1# AND #2 AND #3 | 2379 |
| Cochrane library | depression OR depressive disorder OR depressive disorders OR suicidal OR suicide OR anxiety OR well-being OR wellbeing OR negative emotion OR quality of life OR self esteem OR self-esteem OR mental health OR mental disorders OR mental disorder OR psychological distress OR self efficacy OR self-efficacy OR resilience OR empowerment OR anxiety depression OR depression anxiety OR mood disorders OR depressed individualsOR life skills OR anhedonia OR resilience OR emotional OR psychology OR loss of interest OR reduced energy socialOR participation OR psychological distress OR mental capital OR restless OR psychosocial OR psychiatry | 443,358 |
|  | vitamin D OR vitamin D2 OR vitaminD3 OR D2 OR D3 OR ergocalciferol OR cholecalciferol OR 25(OH)D OR 25 hydroxyvitamin D OR 3 epi 25 hydroxyvitaminD OR calcitriol OR dihydroxycholecalciferol | 1,241,956 |
|  | RCT OR randomized controlled trial OR randomized controlled OR placebo OR placebos | 4,728 |
|  | 1# AND #2 AND #3 | 3011 |

**Sensitivity analysis plot**


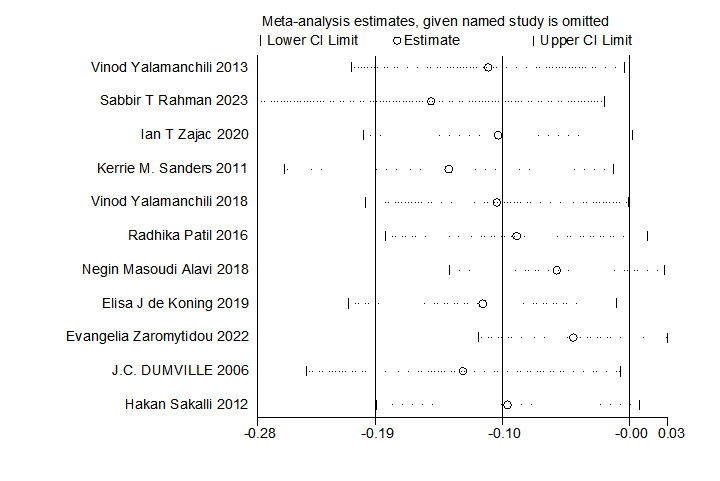

Supplement: Supplementary file 1 [file Table_1.docx]
